# The Importance of Murine Models in Determining In Vivo Pharmacokinetics, Safety, and Efficacy in Antimalarial Drug Discovery

**DOI:** 10.3390/ph18030424

**Published:** 2025-03-18

**Authors:** Glory Adebayo, Opeyemi I. Ayanda, Matthias Rottmann, Olusola S. Ajibaye, Gbolahan Oduselu, Julius Mulindwa, Olayinka O. Ajani, Oluwagbemiga Aina, Pascal Mäser, Ezekiel Adebiyi

**Affiliations:** 1 Covenant University Bioinformatics Research (CUBRe), Covenant University, Ota PMB 1023, Nigeria; glory.adebayopgs@stu.cu.edu.ng (G.A.); gbolahan.oduselu@covenantuniversity.edu.ng (G.O.); ola.ajani@covenantuniversity.edu.ng (O.O.A.); 2 Department of Biological Sciences, College of Science and Technology, Covenant University, Ota PMB 1023, Nigeria; 3 Biochemistry and Nutrition Division, Nigerian Institute of Medical Research, Yaba PMB 2013, Nigeria; sajibaye@yahoo.com (O.S.A.); gbengaaina2003@yahoo.com (O.A.); 4 Swiss Tropical and Public Health Institute, Kreuzstrasse 2, CH-4123 Allschwil, Switzerland; matthias.rottmann@swisstph.ch (M.R.); pascal.maeser@swisstph.ch (P.M.); 5 Department of Chemistry, College of Science and Technology, Covenant University, Ota PMB 1023, Nigeria; 6 Department of Biochemistry and Sports Science, College of Natural Sciences, Makerere University, Kampala P.O. Box 7062, Uganda; julius.mulindwa@mak.ac.ug; 7 Division of Applied Bioinformatics, German Cancer Research Center (DKFZ), 69120 Heidelberg, Germany; 8 African Centre of Excellence in Bioinformatics & Data Intensive Science (ACE), Kampala P.O. Box 7062, Uganda; 9 Infectious Diseases Institute, Makerere University, Kampala P.O. Box 22418, Uganda

**Keywords:** antimalaria, drug discovery, efficacy assessments, murine models, pharmacokinetics

## Abstract

New chemical entities are constantly being investigated towards antimalarial drug discovery, and they require animal models for toxicity and efficacy testing. Murine models show physiological similarities to humans and are therefore indispensable in the search for novel antimalarial drugs. They provide a preclinical basis (following in vitro assessments of newly identified lead compounds) for further assessment in the drug development pipeline. Specific mouse strains, non-humanized and humanized, have successfully been infected with rodent *Plasmodium* species and the human *Plasmodium* species, respectively. Infected mice provide a platform for the assessment of treatment options being sought. In vivo pharmacokinetic evaluations are necessary when determining the fate of potential antimalarials in addition to the efficacy assessment of these chemical entities. This review describes the role of murine models in the drug development pipeline. It also explains some in vivo pharmacokinetic, safety, and efficacy parameters necessary for making appropriate choices of lead compounds in antimalarial drug discovery. Despite the advantages of murine models in antimalarial drug discovery, certain limitations are also highlighted.

## 1. Introduction

The Global Technical Strategy for Malaria has maintained a responsibility to facilitate the global eradication of malaria through salient measures [1]. Major drivers of malaria elimination are the control efforts galvanized into achieving great strides in the global eradication pursuit [2]. Global malaria control efforts rely on a combination of modern diagnostic tools, preventive strategies, and effective treatment measures [2]. As a key factor of the treatment measure, the effectiveness of existing medications has seen a decline over the years as a result of drug resistance [3]. This has posed a notable challenge and has influenced unrelenting continuous efforts towards the discovery and development of new antimalarial drugs [4]. The discovery and development of new antimalarial entities focus on evading the drug resistance hurdle and embracing increased effectiveness with suitable dosage regimens [5]. Novel drug entities are also being discovered based on specific targets and newly identified mechanisms of action [5,6]. Potent novel entities are prioritized based on their fast antimalarial action and capacity to evade drug resistance hurdles as determined by their pharmacodynamic and pharmacokinetic profiles [7].

**Figure 1:**
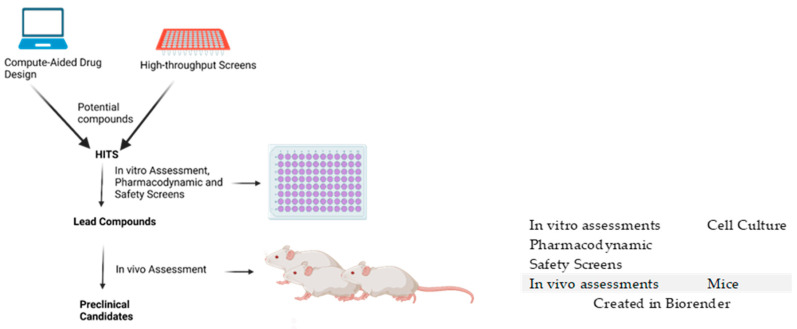
Antimalarial drug discovery workflow.

Due to the significant amount of time required to begin the antimalarial drug discovery process from natural sources, modern antimalarial drug discovery has employed computational and technological tools to develop computer-aided potential antimalarial compounds by virtual screening as an initiation into the drug discovery pipeline [7,8]. The discovery of new chemical entities through high-throughput screening of thousands of potentially active compounds is an early step into the drug discovery pipeline [7,8,9]. In silico-based predicted ligands are prioritized based on their predicted binding affinity to specific target proteins and the molecular dynamics associated with the ligand interactions [10]. Predicted pharmacokinetic and physicochemical properties aid in filtering through identified potential compounds that may be active early lead compounds [8]. Early leads then undergo optimization procedures, and the successful ones are further analyzed. Once optimized and passing all required in vivo tests as outlined by the Medicine for Malaria Ventures (MMV), the resulting compounds may proceed as antimalarial preclinical candidates (Figure 1) [5].

Computer-aided drug design and high-throughput screens are necessary to identify specific potent compounds (hits) from a library of potential compounds. Selected hits undergo rigorous in vitro assessments including pharmacodynamic and safety screens. Compounds showing the best activities following the in vitro assessments may become lead compounds that undergo in vivo screenings, such as antimalarial efficacy, pharmacokinetics, and toxicity assessments. Compounds showing the best activities move on to become preclinical candidates.

Animal models (in vivo) (Table 1) are part and parcel of the antimalarial drug discovery venture [5]. Hamsters (*Mesocricetus auratus*), mice (*Mus musculus*), rats (*Rattus* species), dogs (*Canis lupus familiaris*), and macaques (*Macaca* species) are commonly employed as animal models during the drug discovery process. Murine models are currently being explored in other areas of malaria research besides drug discovery, emphasizing their indispensable utility. Humanized mice are primarily used during efficacy studies in the drug discovery process [5,11,12]. The selection of an animal model for in vivo evaluations depends on the degree of similarity between the target protein in the rodent and the human parasite [13]. These animal models are necessary for predicting drug responses in humans [14]. In addition, murine (mice) models are considered more accessible and versatile, hence their preference over other animal species in early drug discovery [13,15].

Despite the advantages of the murine model, alternative preclinical models can be explored in the absence of mice. Macaques have been used in the evaluation of efficacy, pharmacokinetics, and toxicity of potential antimalarials in drug discovery [16,17]. They have been leveraged as an experimental model in malaria vaccine development [18,19]. *Macaca species* are primarily inoculated with *Plasmodium knowlesi* [20]. Hamsters were successfully inoculated with *P. berghei* malaria [21,22], and several studies have been conducted in the treatment of *Leishmania* species or *Schistosoma* species in hamsters using antimalarial drugs [23,24]. This suggests that hamsters may be an eligible experimental model for drug screening in antimalarial drug discovery. Dogs are commonly and primarily used to determine the pharmacokinetic and toxicity properties of lead compounds in the antimalarial drug discovery process [25,26]. *Rattus* species are commonly used to determine the efficacy, pharmacokinetic, and safety properties of lead compounds in antimalarial drug discovery [27,28].

**Table 1:** Experimental models used in antimalarial drug discovery.

Experimental Models	Study Type in Antimalarial Drug Discovery	Plasmodium Species Inoculated	References
*Macaca species*	Efficacy, Pharmacokinetics, Toxicity	*Plasmodium knowlesi*	[16,17,20]
*Mesocricetus auratus*	-	*Plasmodium berghei*	[21,22]
*Canis lupus familiaris*	Pharmacokinetics, Toxicity	-	[25,26]
*Rattus species*	Efficacy Pharmacokinetics, Toxicity	*Plasmodium berghei*	[27,28]

The discovery of new antimalarials employing murine models for investigative purposes, including efficacy, vaccine development, and safety, dates back decades [29]. Human physiology and genetics are closely related to murine models, providing many opportunities to explore malaria research [30]. Humans and mice share 99% similar conserved regions of their genome [31]. Understanding murine responses in host–pathogen interactions during malaria infection or drug discovery provides insights into the possible parallel outcomes in humans, especially when murine strains are passaged with the susceptible rodent *Plasmodium* strains [30]. Genetic engineering is also used to manipulate rodents to produce transgenic strains. Humanized mice models are the outcomes of these manipulations; they are, therefore, beneficial to different experimental designs in malaria research [32]. Despite the similarities between human malarial infection and malaria infection in murine models, there are specific differences, such as *P. berghei* hepatocytic development occurring faster than *P. falciparum* hepatocytic development [33,34] and the presence of two thiamin biosynthesis enzymes that participate in decreasing the parasite’s proliferative capacity in *P. falciparum,* which is absent in *P. berghei* [35]; Kelch 13 (associated with artemisinin resistance) protein is constituted of 726 amino acids in *P. falciparum* but 738 are found in *P. berghei* among other differences [36]. These and other differences could hinder the absolute application of results obtained from mice malaria research in human malaria research.

Some of the advantages of murine models over other animal species used in antimalarial drug discovery include the following:

1. Mice genes can be manipulated [30].
2. Tissue sections are accessible for examination in case of histopathology [13,37].
3. This animal model is miniature-sized and can be handled easily [38].
4. Mouse models are affordable [38].

The rodent malaria Plasmodium species are *Plasmodium berghei*, *Plasmodium chabaudi*, *Plasmodium vinckei*, and *Plasmodium yoelii*. *P. berghei* ANKA strain infects murine models and is used as a model for *P. falciparum* infection in humans [39]. *P. berghei* ANKA and *P. yoelii* 17XL confer lethality during infection in mice and, therefore, serve as a model for severe malaria [40]. Meanwhile, 17XNL *P. yoelii* confers non-lethality and has been used as a model in malaria vaccine development [41]. *P. chabaudi* provides a great representation of the human malaria pathology and immunology. *P*. *vinckei* exists as both lethal and non-lethal strains [42]. Overall, the rodent malaria models are more straightforward to use during drug discovery than during vaccine development, as it is easier to protect mice from malaria infection than humans [43].

## 2. Current Murine Models Used in Antimalarial Drug Discovery

Most murine strains used in malaria research are inbred strains. The reason for using inbred strains is the uniformity obtained from individual experimental outcomes, especially in antimalarial drug discovery [44,45]. Overcoming some of the phylogenetic distance between mice and humans is the rationale for using humanized mouse strains [32].

Mice are either susceptible or resistant to *Plasmodium* infections. Their resistance may indicate non-lethality of the parasite to mice or late mortality [46]. Screening of novel compounds that consider the fatality of cerebral malaria also requires the use of murine models susceptible to cerebral malaria pathogenesis [47]. Different mouse strains important to antimalarial drug discovery are highlighted in this review.

### 2.1. A Summary of the Various Inbred Mice That Have Been Used and Their Strengths and Weaknesses

Various inbred strains of mice have been used in malaria research. *Plasmodium*-infected Bagg Albino c (BALB/c) mice are widely used in malaria research. Some of such studies include understanding the gut microbiome and its relationship with malaria pathogenesis, vaccine development, the discovery of new chemical entities in ethnomedicine, and more [48,49,50]. BALB/c mice are photophobic and possess a lengthy reproductive threshold [51,52]. Mortality in these mice is delayed until high parasitemia is reached, and they are resistant to cerebral malaria [32,53]. These mice can be infected with the different rodent *Plasmodium* species and can be used in efficacy studies of new chemical entities for antimalarial drug discovery (Table 2). BALB/c mice show anxiety, depression, and aggression naturally. Their immune system is not a representation of the human immune system [54].

AKR/J inbred mice are resistant to *P. berghei* infection; therefore, they are rarely considered as models for in vivo antimalarial efficacy testing in drug discovery [55,56]. This mouse strain displays resistance to cerebral malaria due to its deficiency in the complement component C5 [57]. Nevertheless, AKR/J mice have better visual capacity than other albino mice despite aging [58].

C3H/HeJ mice are an immunocompetent inbred strain susceptible to both *P. berghei* and *P. chabaudi* malaria. They have displayed reduced responses to chloroquine treatment but efficacious responses to dihydrotriazines and biguanides in the treatment of babesiosis and malaria [59,60,61]. This strain of mice is more commonly associated as a model for babesia research than malaria [62]. C3H/HeJ mice possess immunosuppressive capacity; therefore, their disease severity is low [63].

CBA mice are an inbred strain susceptible to *P. berghei* infection. They are commonly used as a model for cerebral malaria because they are genetically predisposed to it at hypoparasitemic conditions even when infected with other rodent strains including *P. chabaudi* and *P. yoelii* [55,64]. CBA mice possess a homogenous genetic characteristic and uniformity in their response physiologically. They have a short life-span (6 months) due to an inherited foam cell reticulosis [65].

SJL/J is an inbred mouse strain susceptible to *P. berghei* and *P. chabaudi. P. berghei* infection may lead to severe malaria and, consequently, cerebral malaria but in an asymptomatic condition [47,66]. This strain may be considered as a murine model for the screening of lead compounds in antimalarial drug discovery. SJL/J is resistant to *Cryptococcus* infection. Despite this, this mouse strain can briefly represent malaria disease states in humans.

C57BL/6 mice are commonly used inbred strains in antimalarial drug discovery programs. They have a competent immune system, and they are also called ‘B6” [54,67,68,69]. C57BL/6 mice were successfully inoculated with *P. falciparum*. Hence, they are recommended for use in antimalarial drug discovery [67]. They are also widely used as a model of human cerebral malaria and immunological responses to malaria. They are resistant to *P. chabaudi* malaria [32,70,71,72]. Nevertheless, similarities occur between the diseased (C57BL/6) mice model infected with *Plasmodium berghei* ANKA and the human disease [73]. Essentially, sequestration of *P. berghei* occurs in the brain of the rodent as a similar condition occurs during pathogenesis in the human disease [73].

DBA/2J mice are inbred strains rarely used as models in antimalarial drug testing, but they have been explored for neurological experiments. However, they are resistant to cerebral malaria but susceptible to *P. berghei* infection [53,55,59,74]. This mouse strain has also been infected with other rodent parasite strains, including *P. yoelii*, *P. chabaudi*, and *P. vinckei*, making it suitable for drug screening [55,59]. DBA/2J mice display some heterogeneity in their genetic constitution [75].

### 2.2. Outbred Mice

Swiss Webster is a widely used outbred mouse susceptible to *P. berghei*, *P. chabaudi*, and *P. yoelii* infection and is currently still used in the determination of the in vivo efficacy of new chemical entities [76]. This outbred strain also manifests cerebral malaria in severe parasitemia cases, which is also considered in treatment development [77]. Transgenic parasite lines of *P. berghei* have also been explored in this mouse model [78].

The Institute of Cancer Research (ICR) mice are outbred and highly susceptible to *P. chabaudi* infection as they are to *P. berghei* [72,79]. They are commonly used as a model for the in vivo screening of potential antimalarial molecules obtained from plant sources [80,81,82]. These mice also manifest cerebral malaria in severe cases [47].

CD1 mice are Swiss-based outbred strains susceptible to multiple rodent *Plasmodium* species [12]. This mouse strain is employed to evaluate the pharmacokinetic properties and the safety of novel compounds [12,83].

Naval Medical Research Institute (NMRI) mice are outbred strains susceptible to *P. berghei* infection [84,85]. In NMRI mice, *P. berghei* parasite development occurs exponentially. Therefore, the efficacy of new chemical entities can be estimated suitably in this mouse model [85]. Hepatic-stage *Plasmodium* infection is also assessed using this model, which could mean the inoculation of transgenic parasites [86]. NMRI mice are also models for cerebral malaria pathogenesis from *P. berghei* or *P. yoelii* infection [47].

### 2.3. Humanized Mice

Humanized mice are now the order of the day in antimalarial drug discovery. They are engrafted with human cells under immunocompromised conditions while also knocking in human genes into the mouse genomes, thereby more effectively representing human parasite pathogenesis for treatment purposes [87]. Although non-humanized mouse models have aided in successful human predictions of the efficacy of new chemical entities and their pharmacokinetics in antimalarial drug discovery, there are certain advantages and disadvantages to using them (Table 3) [73].

NOD/SCID/gamma (c) (null), known as NOG mice, are immunocompromised and void of essential lymphocytes like B and T cells although macrophages remain present [88]. Meanwhile, macrophages have emerged as critical natural protective agents in malaria pathogenesis in humans, which can now be assessed in this mouse strain [89]. These human model mice can be infected with human *Plasmodium* parasites for novel therapeutic studies, as well as the evaluation of pharmacological parameters [90].

NOD.Cg-*Prkdc^scid^ Il2rg^tm1Wjl^*/SzJ mouse strain, similar to NOG, is susceptible to both *P. falciparum* and *Plasmodium vivax*. It is helpful for the efficacy determination of new chemical entities against *P. falciparum.* Efficacy can also be evaluated against liver-stage hypnozoites characterized by *P. vivax* infection [91,92].

The FRG NOD huHep mouse is a model for the human chimeric liver in *P. falciparum* malaria research. It has aided the understanding of *P. falciparum* pathology transiting from hepatic- to erythrocytic-stage development, which presents a platform for novel antimalarial screening [93].

5xFAD mice are a transgenic mouse model that is predisposed to Alzheimer’s disease and they are characterized by the presence of some amyloid plaques [94]. Cerebral malaria has been hypothesized to be associated with apolipoprotein-mediated amyloidosis, whose pathogenicity may also be observed in Alzheimer’s disease [95]. Elevated levels of apolipoprotein are observed in the mouse brain during cerebral malaria pathogenesis, which could result in neurological dysfunction, as was observed in mice [95]. Artesunate is reported to alleviate the amyloidosis pathology in 5xFAD mice [96]. This suggests that 5xFAD mice could be explored for antimalarial drug development in treating cerebral malaria.

A future perspective of murine models in antimalarial drug discovery is that although humanized mice have been able to recapitulate the malarial efficacy in humans, the extrapolation of pharmacokinetic responses from mice to humans needs to be clearly understood for all drugs and their mechanism of action [97]. This means that we need to know if every antimalarial can clearly represent the pharmacokinetic parameters in humans, using humanized mice [97].

**Table 2:** Murine strains used in antimalarial drug discovery.

Murine Model	Type	Preclinical Assays Conducted	Resistant/Susceptible to *P. berghei*	*Plasmodium* Species Assessed	References
BALB/c	Inbred	Efficacy, Pharmacokinetics, Safety,	*Susceptible*	*P. yoelii*, *P. chabaudi*, *P. vinckei*	[46,50,59,98,99,100]
AKR/J	Inbred	Pharmacokinetics	Resistant	-	[58,59]
C3H/HeJ	Inbred	Efficacy	Susceptible	*P. chabaudi*	[59,61,63]
CBA	Inbred	Efficacy	Susceptible	*P. yoelii*, *P. chabaudi*, *P. vinckei*	[55,64,65,101,102]
SJL/J	Inbred		Susceptible	*P. chabaudi*	[59,66,103]
C57Bl/6	Inbred	Efficacy, Safety	Susceptible	*P. yoelii*, *P. chabaudi P. falciparum*, *P. vinckei*	[32,55,70,71,72].
DBA/2J	Inbred		Resistant	*P. yoelii*, *P. chabaudi*, *P. vinckei,*	[75,104,105,106,107,108]
Swiss Webster	Outbred	Efficacy, Toxicity	Susceptible	*P. yoelii* *P. chabaudi*	[109,110,111,112,113]
ICR	Outbred	Efficacy, Pharmacokinetics, Safety	Susceptible	*P. yoelii,* *P. chabaudi, P. vinckei*	[12,114,115,116,117]
CD1	Outbred	Efficacy, Pharmacokinetics, Safety	Susceptible	*P. chabaudi, P. yoelii*	[47,84,85,86,118]
NMRI	Outbred	Efficacy, Pharmacokinetics, Safety	Susceptible	*P. chabaudi, P. yoelii*	[90,119,120,121,122]
NOD/SCID/γcnull (NOG)	Humanized	Efficacy, Pharmacokinetics		*P. falciparum*	[91,92,104]
NOD.Cg-*Prkdc^scid^ Il2rg^tm1Wjl^*/SzJ	Humanized	Efficacy, Pharmacokinetics		*P. falciparum*, *P. vivax*	[123,124]
FRG NOD huHep	Humanized	Efficacy		*P. falciparum*	[96]
5xFAD	Transgenic	-	-	-	

**Table 3:** Advantages and disadvantages of using non-humanized and humanized mouse models in antimalarial drug discovery.

Advantages of Using Non-Humanized Mouse Models in Antimalarial Drug Discovery	Disadvantages of Non-Humanized Mice Models in Antimalarial Drug Discovery	Advantages of Humanized Mice Models in Antimalarial Drug Discovery	Disadvantages of Humanized Mice Models in Malaria Research
Despite the challenge of an unclear representation of the human immune system in mice, basic immunological responses have been used to give reliable results of predicted failures in malarial vaccine development [73].	Immunology in non-humanized mice is not a clear representation of human immunology [73].	Enhancement of potential antimalarial efficacy studies has been granted through the use of humanized mice models [125].	Mouse innate immunity is difficult to reduce to increase human adaptive immunity [126].
Gaining insight into cerebral malaria is technically challenging due to the inaccessibility of the human brain tissue.		Pharmacodynamic/pharmacokinetic studies of novel compounds can be conducted to obtain a representation of what could be obtained in humans [125].	It is challenging to engraft human cells into mice while generating humanized mice [127].

## 3. In Vivo Pharmacokinetic (PK) Studies

**Figure 2:**
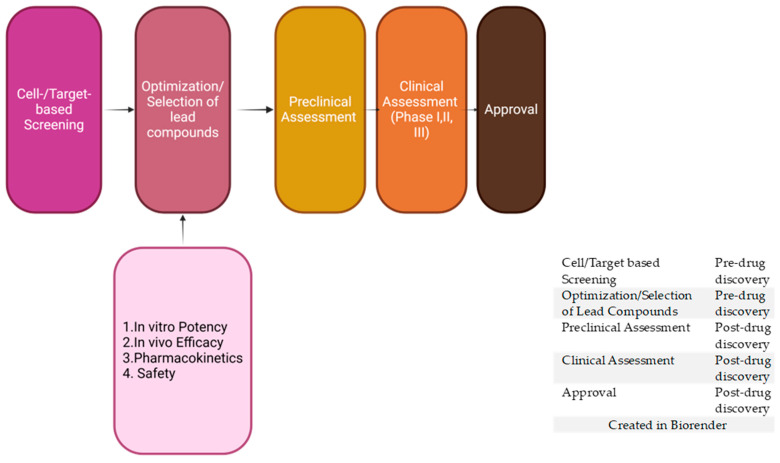
Drug development pipeline.

Several factors, including excellent pharmacokinetic properties, increased half-life, and appropriate metabolic distribution, can influence the potency of a compound [128]. Pharmacokinetic properties such as in vivo bioavailability are considered a significant complement to the in vitro efficacy assessment in the identification of new leads in antimalarial drug discovery [129] (Figure 2). Drug metabolism and pharmacokinetic (DMPK) profiling is essential to the optimization of potent compounds in the antimalarial drug development program, and it determines how far these compounds can traverse the drug discovery pipeline [130]. DMPK profiling involves both in vitro and in vivo experiments. In vitro experiments require the use of cells in a controlled environment, but in vivo experiments require the use of animal models, of which murine models are critical [130].

### 3.1. Oral Bioavailability

**Figure 3:**
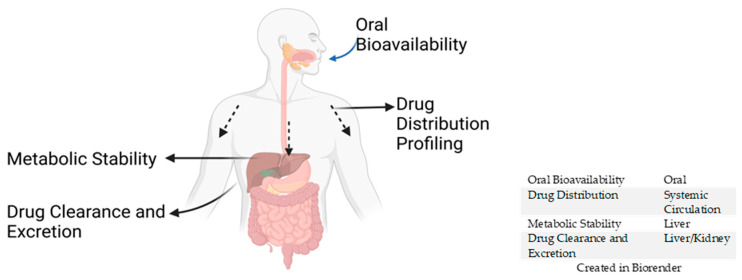
In vivo pharmacokinetic parameters in antimalarial drug discovery.

In vivo, oral bioavailability of drugs is associated with oral absorption, and oral bioavailability in rodent models may be used predict that in humans [131,132]. Oral bioavailability is the percentage of administered test compound that ends up in the bloodstream (Figure 3). In contrast, oral absorption is the amount of test compound taken up by the gastrointestinal system [132,133]. Oral bioavailability influences the decision on the drug exposure and dosage regimen of test compounds. Low oral bioavailability may lead to the failure of lead compounds despite in vitro potency, which must be prevented in clinical trials to avoid the wastage of resources [134,135]. Although several in silico tools have been designed to predict oral bioavailability, they do not always correlate with actual in vivo experiments [132,134]. Nevertheless, predictive tools can guide the decision processes for potent test compounds’ experimental oral bioavailability determination that should be selected as acceptable lead compounds [132,134]. In vivo, oral bioavailability is usually estimated from calculations after the experiment has been conducted in rodent models using high-performance liquid chromatography–electrospray ionization–tandem mass spectrometry (HPLC-ESI-MS/MS) [132,136]. The oral bioavailability of a test compound can be elevated by increasing the solubility and reducing the compound’s melting point [135,136,137]. Excellent oral bioavailability represents good drug gastrointestinal permeability and absorption [135,136].

In the selection process of potent hits traveling from phenotypic screening, it is of essence to first consider their in vivo pharmacokinetic profile [138]. If the pharmacokinetic properties are undesirable due to challenges occurring from oral bioavailability, other routes of administration are best employed, such as the intravenous route [138].

Oral bioavailability is associated with the oral absorption of the test compounds. Drug distribution profiling evaluates how the test compounds travel the systemic circulation. Metabolic stability is a measure of the metabolic rate of the test compounds in the liver. Drug clearance and excretion describes the elimination rate of the test compound from the liver.

### 3.2. Drug Distribution Profiling

In vivo, drug distribution is determined by evaluating how barriers such as the blood–brain barrier (BBB) are penetrated and how the volume of compounds that traverse the membrane to reach their target is measured with respect to plasma protein binding (PPB) [139,140]. Drug distribution is a reversible process in pharmacokinetic profiling that begins with the process of drug dilution to plasma binding through absorption, and then drug molecules are dispersed to other parts (Figure 3) [141].

The volume of distribution (Vd) of a test compound is the apparent volume of the test compound in plasma and it measures the extent of distribution of the compound [138,142,143]. The Vd is very important because it influences the half-life of the test compound as well as the dosage frequency [142]. When the Vd of a test compound is low, the half-life of that compound will be low [144]. The apparent volume of distribution of a test compound is a required pharmacokinetic property when a decision is to be made on the choice of lead compounds, especially if they are considered in combination with other compounds or existing antimalarials such as artemisinin-based treatment [139,145,146]. The apparent volume of distribution is usually determined after intravenous administration [147,148]. Dosage optimization of drugs is also influenced by the Vd, among other pharmacokinetic parameters [149].

Drugs or test compounds are either bound to plasma proteins or lipids or unbound. When bound in the blood, they are bound to proteins and lipid molecules, hence called plasma protein binding (PPB) [150]. Determining PPB for lead compounds is critical to inform further decisions in the drug development pipeline. If it is reversible, PPB does not influence the efficacy of the test compound in vivo [151,152]. High PPB means that the apparent volume of distribution and lipophilicity is high. Still, the elimination of the test compound is reduced, which is advantageous in the context of the extended half-life [141,153,154,155]. The increased half-life of lead compounds may be traced to high PPB. It influences the raising of the apparent volume of distribution and may prosper the course of a single-dose regimen of the lead compound being validated in vivo [154]. PPB can be assessed in vivo in rodents using ultracentrifugation or equilibrium dialysis [154,156].

Albumin and alpha-1-acid glycoprotein (AAG) are two common plasma proteins measured when evaluating the PPB property of a test compound [144,155]. Both plasma proteins are produced in the liver, and plasma albumin exists in higher concentrations than plasma AAG. Abnormal physiological conditions, therefore, alter the functioning of these plasma proteins [153,155]. Albumin in plasma binds to both acidic and basic compounds. Meanwhile, AAG binds to more basic, neutral lead compounds [153]. It is worth noting that children and pregnant women display reduced levels of plasma protein binding to albumin. Therefore, the apparent volume of distribution will be reduced, and this should be considered in the drug development plan for neonates [149,153,157].

#### BBB Dysfunction and Cerebral Malaria

BBB penetration is an important in vivo pharmacokinetic parameter for consideration during antimalarial drug development. BBB penetration is a parameter for the representation of the drug distribution profile of test compounds [140,158]. It is a measure of the concentration of test compounds present in the brain against the concentration found in the blood [140]. BBB is of particular interest when treating cerebral malaria. A dysfunctional BBB is significantly associated with cerebral malaria [159]. *Plasmodium* parasites compromise the BBB, leading to neurological dysfunction. Certain markers that escape through the compromised BBB can be traced when found in the blood, for instance, the “tau” protein [160]. Experimental cerebral malaria thrives in murine models on the basis that infected erythrocytes are amassed in the brain’s vascular system [161]. Artesunate is currently recommended as a treatment for cerebral malaria in the current mouse model (C57BL/6) used for cerebral malaria infected with *P. berghei*. [162,163]. In vivo BBB integrity is determined through spectrophotometry, having extracted the brain from experimental mice injected with Evans blue [159,162]. Intravenous administration of lipid-carrying potential drug molecules could elevate BBB integrity [163]. Intranasal delivery of nanostructured lipid carriers has also been reported to effectively traverse the blood–brain barrier for treatment for cerebral malaria [163,164].

### 3.3. Metabolic Stability

Microsomal stability assay is a test conducted to evaluate the metabolism rate of lead compounds undergoing optimization. It measures the metabolic stability of the test compounds in both in vitro and in vivo hepatocytes [165]. The metabolism of test molecules is usually a function of hepatic processes facilitated by liver cytochrome enzymes [166].

Microsomes possess cytochrome P450 enzymes and enzymes like uridine 5′-diphospho-glucuronosyltransferase that metabolize most antimalarials in mice liver [109,167]. Microsomal stability of several potential antimalarials has been tested in rats, mice, dogs, and human microsomes [68,168]. The metabolic rate of microsomal enzymes in *Plasmodium*-infected mice is reported to be lower than in the uninfected counterparts, and the measurement of how low this clearance would be is yet unclear [109]. Metabolic stability is measured as a function of the half-life of the test compounds, the level of liver microsome proteins present, and the intrinsic clearance (in vitro) [109,168]. The half-life is the removal of 50% of the test compound, while intrinsic clearance describes the hepatic activity (as a function of the microsome protein content) against the test compound minus the influence of other hepatic factors such as the blood flow in the liver [169]. During the assay, samples of the test compound in the presence of extracted liver microsomes are collected at different time points for the estimation of intrinsic clearance [170]. Increased clearance rates insinuate decreased half-lives of the test compounds in the liver, indicating a large volume of drug distribution [168,171]. Microsome stability assays are evaluated for blood-stage, liver-stage, and transmission-blocking potential antimalarials [172,173,174,175]. Lead compounds displaying excellent metabolic stability proceed further in the antimalarial drug development pipeline [175]. In vitro microsomal stability assays are conducted more frequently than in vivo microsomal assays in antimalarial drug development [170,172,173,174,175,176].

### 3.4. Drug Clearance and Excretion

The rate at which a test compound is eliminated from the animal describes excretion. Meanwhile, the total clearance involves the disappearance of the drug molecules from the plasma–compound-bound complex at a given time [177]. The metabolic activities in the liver and kidney marshal this plasma clearance (Figure 3). Clearance from the liver is termed ‘hepatic intrinsic clearance’, and from the kidney, it is called ‘renal clearance’ [9,166]. In vitro, hepatic intrinsic clearance of free unbound molecules is evaluated using liver microsomes. High intrinsic clearance is directly proportional to a rise in the octanol/pH 7.4 buffer partition coefficient. The partition coefficient is the distribution of a solute’s concentration between the oil and water phases [178]. Reduced levels of intrinsic clearance of unbound molecules increase the half-life of the molecules [154,166]. This intrinsic clearance provides a basis for the prediction of the in vivo total clearance of the test molecule [166]. Data generated from the in vitro hepatic intrinsic clearance are used to make predictions for the in vivo hepatic intrinsic clearance. The transition from in vitro to in vivo hepatic intrinsic clearance requires the use of scaling factors that consider the weight of the in vivo mammalian species to be used [166].

The excretion of compounds undergoing optimization as a pharmacokinetic property incorporates the half-life or elimination of the drug in vivo [177]. Elimination of a test compound is the permanent removal of the compound from systemic circulation [179]. Renal excretion provides the means for eliminating unbound test molecules and can be evaluated in vivo in rodent models [68]. Renal clearance is facilitated by glomerular filtration and active transport (both can be extrapolated using the rodent model for evaluation) [180].

## 4. In Vivo Safety Studies

**Figure 4:**
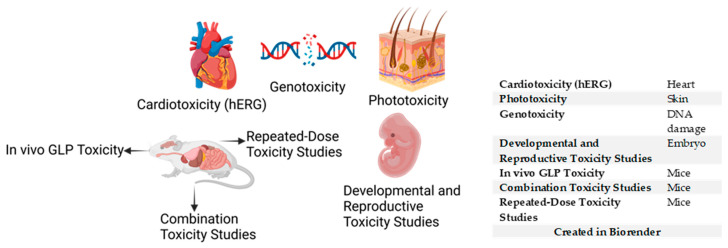
In vivo safety parameters in drug discovery.

Toxicity outcomes of the potent molecules must be considered at an early stage of the drug discovery program using preclinical testing such as mouse models [125] (Figure 4). Hewitt et al. [125] provided a guideline for safety tests to be conducted on lead compounds in vivo before further drug development. Some in vivo toxicity assays include cardiotoxicity, genotoxicity, phototoxic potential, Good Laboratory Practice (GLP) toxicology studies, combination toxicity studies, cumulative exposure studies, and developmental and reproductive toxicology testing [5,125,181].

### 4.1. Cardiotoxicity (hERG)

Cardiotoxicity as a detrimental side effect has been associated with certain classes of antimalarials, including quinolines. This is a critical subject as new molecules are being investigated for antimalaria development [182,183]. At an early stage of preclinical studies, assessing the toxic effects of lead compounds on cardiomyocytes is recommended (World Health Organization) for investigation with measurements of the molecular marker human ether-ago-go-related gene (hERG) [5,125,182,183]. Siqueira-Neto et al. [5] recommend an absence of toxicity against hERG at 1 µM during the early stage of preclinical tests. Meanwhile, at the late stage, the threshold for hERG toxicity must be further reduced when evaluated in vivo. In vivo, cardiotoxicity of antimalarials and potential antimalarials have been determined in rats and zebrafish [183,184].

### 4.2. Genotoxicity

Genotoxicity assays are designed to measure the level of genetic damage conferred by a test molecule, and the odds of the damage caused are at risk of transmission from one generation to another. There are both in vitro and in vivo assays conducted to determine the genotoxic status [185,186]. It is essential to conduct in vivo genotoxicity experiments in addition to in vitro experiments in order to obtain reliable results [186]. Drug candidates with genotoxic potential are excluded during early preclinical screening [186,187]. In vivo genotoxicity has been conducted in rodent models and dogs in the early stages of antimalarial drug discovery [181,188]. Carcinogenicity studies are not considered during antimalarial drug discovery because the dosage regimen is for a short period [125].

### 4.3. Phototoxicity

Phototoxicity occurs when undesirable skin responses are observed after administering a drug candidate or a test molecule [189,190]. The phototoxic potential of antimalarial drug candidates has recently been considered for assessment during an early preclinical phase. This test is also conducted in vivo [5,125,191]. In phototoxicity testing, mice are treated with test compounds by oral administration, after which the mice are irradiated with ultraviolet light, which is a simulation of sunlight. Phototoxic reactions are then observed [192]. For instance, compounds with the best potencies displayed phototoxicity while the Second World War was in progress, which led to halting the compounds’ development [146]. Pyrimethamine monotherapy is reported to display phototoxicity in humans when exposed to ultraviolet A or B light, which results in damaged cells [193].

### 4.4. Good Laboratory Practice (GLP) Toxicology Studies

Good Laboratory Practice (GLP) toxicology assessment is a regulatory laboratory procedure conducted in vivo (in rodents and dogs) for 2 weeks [125,194]. This toxicity study is a dose range-finding experiment in rodents to determine how harmful specific concentrations of the lead molecule will be according to GLP standards [195]. One frequently used method is the maximum tolerated dose (MTD) approach, which assesses the toxicity and dose range of the potential drug compound [195,196]. GLP procedures are primarily non-clinical studies conducted before clinical studies in drug development [125,195].

### 4.5. Combination Toxicity Studies

Combination therapy in antimalarial drug development is critical to the elimination of malaria due to the challenge of monotherapy resistance [197,198]. In the process of discovering new drug combinations of excellent efficacies, the combination of compounds must not increase the toxicity [199]. Combination toxicity is a non-clinical study conducted for about three months using rodents. Each compound is evaluated individually (and in combination), especially when each compound is exclusively efficacious as a requirement for the combination of test compounds used [200].

### 4.6. Repeated-Dose Toxicity

A repeated-dose toxicology (RDT) study is a non-clinical approach to evaluating the toxicity of the test compounds to determine safety [201]. RDT for potential antimalarials is determined in both rodent and non-rodent species, but particularly in rats, usually for a period of 14 days, where the test compound is administered for all the days [125,202,203]. RDT is a significant testing approach for new chemical entities that are not administered as a single dose, and side effects of the test compounds are sufficiently estimated before further development while considering test compounds with increased half-lives [125,204].

### 4.7. Developmental and Reproductive Toxicity Testing

Developmental and reproductive toxicology is paramount in considering lead compounds that should proceed for further development [205,206,207]. Development and reproductive toxicological studies are preclinical studies conducted to determine the test compound’s embryotoxic or teratogenic effects in vivo, informing the decisions of choice and how to improve the selected lead compounds [5,205,206,208]. Artemisinin displayed embryotoxicity in the first three months in pregnant women [205]. This was observed to be as a result of the time of administration of the compound and the dosage involved. It was, therefore, suggested that women in their first trimester of pregnancy should desist from the use of artemisinin-based therapy [205].

## 5. In Vivo Rodent Efficacy Studies

**Figure 5:**
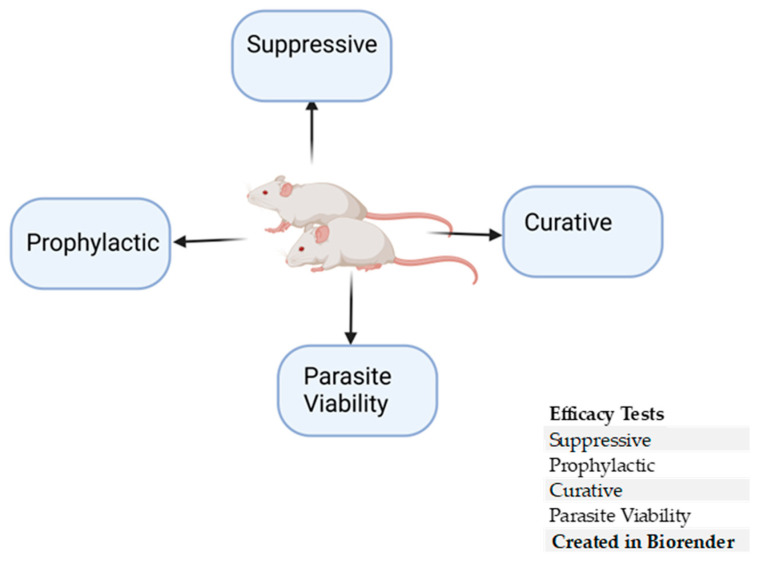
In vivo efficacy parameters in antimalarial drug discovery.

In vivo, efficacy testing in antimalarial drug discovery is an indispensable aspect of the development pipeline (Figure 5) [209]. The potency of lead compounds is evaluated at different developmental stages of the parasite in vivo [5]. Rodents are the face of preclinical efficacy testing in antimalarial discovery, following validations from in vitro screenings and the identification of lead compounds [71]. During efficacy studies, rodent models are selected based on the class of antimalarial candidate being evaluated or the type of population the drug is designed for [209].

Rodent models are amenable to several routes of drug administration. Routes of administration of drug treatments include oral, rectal, intramuscular, intravenous, and subcutaneous. Other routes of administration aside from oral dosage are explored when test compounds are not quickly soluble [129]. The oral route of administration is most common in antimalarial drug discovery and does not require any technical skills. The compound administered essentially undergoes absorption in the small intestine [5,210]. Rectal administration occurs by using the rectum as a route for the drugs; absorption occurs in the rectum and travels to the level [210]. Artesunate has been administered rectally in the case of severe malaria [5]. Intravenous administration occurs by using injection, and compounds can travel through the systemic circulation through the veins [210]. The subcutaneous route of administration is an entry of the drug through the skin, which is also applied through injections at the thigh or buttocks. Drugs are absorbed slowly but sustainably [210]. When compounds are administered through the intravenous or subcutaneous route, *Plasmodium* sporozoites are targeted for clearance [5]. The intramuscular route of administration involves entry through muscles by injections. It requires the technicality of experts and, therefore, should be handled with care [210]. Arteether is administered intramuscularly in the case of severe malaria [211].

### 5.1. Prophylactic Test

A test compound’s prophylactic efficacy is the capacity to prevent the development of the *Plasmodium* parasite, thereby preventing the progression of the parasite’s pathological cycle [212,213]. Prophylactic treatment is administered chiefly to travelers and migrants to and from malaria-endemic countries [212]. In vivo prophylactic test is an immediate efficacy evaluation to be conducted, having pinpointed the lead compound [214]. To evaluate prophylaxis, rodents are initially administered the test compound and inoculated with the rodent *Plasmodium* parasite afterward. The efficacious activity of the test compound is measured by the level of parasite densities or parasitemia at the end of the experiment [215,216]. Lead compounds may be evaluated for their prophylactic efficacy by hindering the parasite development at the pre-erythrocytic/hepatic (causal) or erythrocytic (suppressive) stage [213,217]. Suppressive treatment inhibits erythrocytic-stage parasitic infections [218].

### 5.2. Suppressive Test

The most prevalent in vivo chemo-suppression assay is Peter’s four-day suppressive test, which entails a four-day treatment procedure a few hours after parasite inoculation [71,215]. Mean survival time is also determined during Peter’s suppressive test, which estimates how long the animals survive post-treatment [219]. Parasitemia and percentage suppression are determined from the experiments as a measure of chemo-suppression [215].

### 5.3. Curative Test

Rane’s test is the most widely used to assess the curative efficacy of new chemical entities in vivo [215,220]. During this experiment, treatment of the animals inoculated with the rodent *Plasmodium* parasite begins from day 3 post-infection and lasts for 4 days. Curative treatment is usually administered orally, intraperitoneally, and through other avenues [220]. Curative tests conducted for lead molecules are particularly essential when treating cerebral malaria [220,221].

### 5.4. Parasite Viability

In vivo parasite viability estimation post-treatment has been considered a more informative measure of drug efficacy than just the number of parasites or strength of a reporter signal [222]. Analogous to the concept of colony-forming units in virology, this method uses limiting dilution and in vitro regrowth to determine the number of viable parasites after drug wash out. While this is very laborious and time-consuming, it aids in measuring the lead compound’s cidal effect and the maximal concentration to obtain the optimal cidal effect [222]. Parasite viability as a read-out can correct for underestimation of a drug’s efficacy. For example, artesunate appeared to leave some parasites in circulation after treatment [222]. Nevertheless, these were not actually viable and over-estimation of efficacy was observed with a *P. falciparum* field isolate that appeared to respond to piperaquine but actually would not be cleared by it [223].

### 5.5. Examples of Antimalarial Lead Compounds and the In Vivo Efficacy Assessment Conducted

Some antimalarial leads have demonstrated promising in vivo efficacy, particularly in curative and survivability tests. Some are listed in Table 4.

### 5.6. Limitations to Using Murine Models in Antimalarial Drug Discovery

Phenotypic expressions of malaria are heterogeneous across the different mouse strains. Therefore, it is still unknown which mouse strain manifests the disease best comparable to the human disease [73]. Therefore, it is encouraged that mouse models clearly represent the human disease state [73]. Murine models have been used to predict failed vaccines at the preliminary stage, where these vaccine tests are not always successful [73]. Drugs that lead to anemic consequences in humans do not cause anemia in non-humanized mice during antimalaria treatment [232]. Malaria infection in murine models reduces the integrity of cytochrome P450’s activity in drug metabolism [233]. While conducting in vivo experiments in murine models, the amount of test compound needed is high, which may practically be challenging when applied in human studies [234]. The extrapolation of analyzed results from tests against *Plasmodium berghei* to the human parasite [234].

## 6. Conclusions

Careful selection of lead molecules in preclinical studies is critical to minimizing failures in later clinical trials, where discrepancies between murine and human responses often arise. Therefore, the availability of murine resources in in vivo validation experiments of in vitro-identified lead compounds provides a rich basis for assessing these new chemical entities for their safety, toxicity, dosage regimen, and efficacy in preclinical trials. This informs the Phase I clinical trials and can reduce the risk of failures during clinical trials. Moreover, murine models are beneficial for understanding other malaria-associated bioactivities, for instance, vaccine development, metabolic responses during the disease cycle, and more. These contribute to the malaria control efforts from a broader perspective. In view of this broader perspective, more development in engineering mice towards understanding the disease condition wholly is necessary.

## Figures and Tables

**Table 4:** Some antimalarial leads, with their in vivo efficacy assessment and their murine model.

Lead Compounds	In Vivo Efficacy	Murine Model	Reference
UCF501	Curative	BALB/c/Swiss Webster	[224]
ACT-451840	Curative	NMRI	[225]
WEB-484, WEB-485, WEB-486, WEB-487	Suppressive	NOD-*scid* IL2Rγ^null^	[226]
INE963	Curative	NOD-*scid* IL-2Rγ^null^	[173]
MMV1557817	Suppressive	Balb/c/Swiss Outbred	[227]
AR-42	Curative	BALB/c	[228]
UCT943	Suppressive	NOD-*scid* IL-2Rγ^null^	[229]
Calxinin	Suppressive	C57BL6	[230]
NP046	Suppressive	C57BL/6	[231]

## Data Availability

Not applicable.

## Abbreviations

AAG	Albumin and alpha-1-acid glycoprotein
BALB/c	Bagg Albino
BBB	Blood–brain barrier
GLP	Good Laboratory Practice
hERG	human ether-ago-go-related gene
HPLC-ESI-MS/MS	high-performance liquid chromatography–electrospray ionization–tandem mass spectrometry
ICR	Institute of Cancer Research
PPB	Plasma protein binding
PK	Pharmacokinetic
RDT	Repeat-dose toxicology
TO	Theiler’s Original
Vd	Volume of distribution
